# Regional Differences of Leptospirosis in Sri Lanka: Observations from a Flood-Associated Outbreak in 2011

**DOI:** 10.1371/journal.pntd.0002626

**Published:** 2014-01-16

**Authors:** Suneth B. Agampodi, Niroshan J. Dahanayaka, Anoma K. Bandaranayaka, Manoj Perera, Sumudu Priyankara, Prasanna Weerawansa, Michael A. Matthias, Joseph M. Vinetz

**Affiliations:** 1 Department of Community Medicine, Faculty of Medicine and Allied Sciences, Rajarata University, Saliyapura, Sri Lanka; 2 Tropical Disease Research Unit, Faculty of Medicine and Allied Sciences, Rajarata University, Saliyapura, Sri Lanka; 3 Division of Infectious Diseases, Department of Medicine, University of California San Diego, School of Medicine, La Jolla, California, United States of America; 4 Department of Medicine, Faculty of Medicine and Allied Sciences, Rajarata University, Saliyapura, Sri Lanka; 5 Teaching Hospital, Anuradhapura, Sri Lanka; 6 Instituto de Medicina Tropical Alexander von Humboldt, Universidad Peruana Cayetano Heredia, Lima, Peru; 7 Departamento de Ciencias Celulares y Moleculares, Laboratorio de Investigación y Desarrollo, Facultad de Ciencias, Universidad Peruana Cayetano Heredia, Lima, Peru; University of Washington, United States of America

## Abstract

Leptospirosis is known to be an important cause of weather disaster-related infectious disease epidemics. In 2011, an outbreak of leptospirosis occurred in the relatively dry district of Anuradhapura, Sri Lanka where diagnosis was resisted by local practitioners because leptospirosis was not known in the area and the clinical presentation was considered atypical. To identify the causative *Leptospira* associated with this outbreak, we carried out a cross-sectional study. Consecutive clinically suspected cases in this district were studied during a two-and-a-half-month period. Of 96 clinically suspected cases, 32 (33.3%) were confirmed by qPCR, of which the etiological cause in 26 cases was identified using 16S rDNA sequencing to the species level. Median bacterial load was 4.1×10^2^/mL (inter-quartile range 3.1–6.1×10^2^/mL). In contrast to a 2008 Sri Lankan leptospirosis outbreak in the districts of Kegalle, Kandy, and Matale, in which a predominance of *Leptospira interrogans* serovars Lai and Geyaweera was found, most cases in the 2011 outbreak were caused by *Leptospira kirschneri*. Seven (21.9%) confirmed cases had acute renal failure; five (15.6%) had myocarditis; severe thrombocytopenia (<20,000/uL) was seen in five (15.6%) cases. This outbreak of leptospirosis in the relatively dry zone of Sri Lanka due primarily to *L. kirschneri* was characterized by markedly different clinical presentations and low leptospiremia. These observations and data demonstrate the public health relevance of molecular diagnostics in such settings, possibly related to the microgeographic variations of different *Leptospira* species, but of particular value to public health intervention in what appears to have been a regionally neglected tropical disease.

## Author Summary

Leptospirosis outbreaks occur predictably in Sri Lanka after seasonal rains and flooding in the endemic wet zone. Molecular investigations with quantification of a post-flood leptospirosis outbreak in the non-endemic dry zone of Sri Lanka in 2011 suggest variation of biological, clinical, and molecular characteristics compared to previous reported leptospirosis outbreaks in the endemic areas, probably showing a micro-geographic variation of leptospirosis. This work demonstrates the direct clinical and public health relevance of modern molecular diagnostic technologies to identifying an endemic neglected tropical disease where previously not suspected, especially in the resource-poor setting.

## Introduction

Sri Lanka is a tropical island located southeast of India. Annual rainfall varies from <1,500 mm in “dry” zones, where water reservoirs may dry to completeness, to 5,000 mm in the “wet” zones with a mean annual temperature between 26.5°C to 28.5°C. Based on Ministry of Health notification data, leptospirosis is common in nine of 24 districts in Sri Lanka with annual disease incidence rates varying from 31 to 164/100,000 population [1]. All leptospirosis endemic districts are within wet zones and have an annual rainfall in excess of 2,000 mm. In wet zone districts, paddy farming activities, high rainfall, moist soil, year-round water retention in paddy fields, the use of buffalo in agriculture and peri-domestic animal farming in rural areas provide ideal environments for the transmission of leptospirosis.

**Figure 1:**
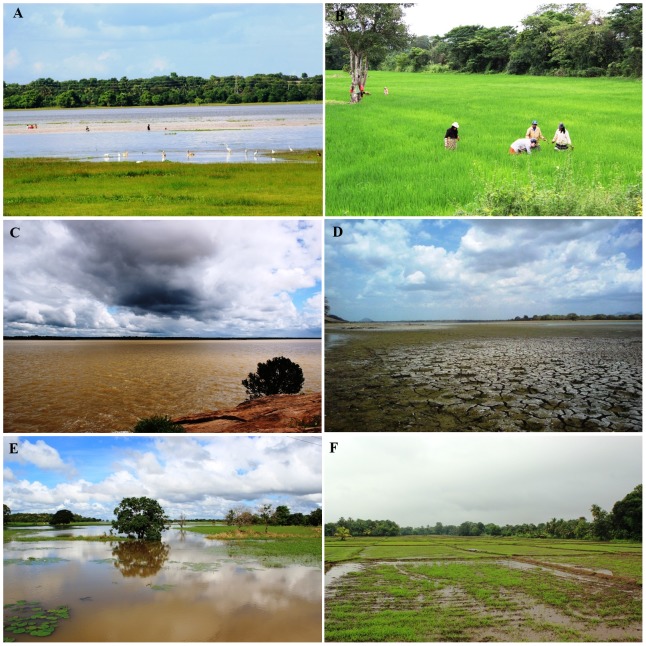
Geography of Anuradhapura showing paddy fields and water reservoirs. (A)Partially dried water reservoir (Nuwara Wewa) during dry season (B) Females removing weeds in paddy fields (C) Water reservoir during the rainy season (D) Dried up reservoirs during dry season (E) Flooding in Anuradhapura (F) Paddy field during the cultivation season.

In 2011, a large outbreak of leptospirosis was observed in Anuradhapura district of Sri Lanka, which was not previously classified as an endemic area for leptospirosis. Anuradhapura is located in North Central province of Sri Lanka, in the dry zone of the country. The annual rainfall is 1,200–1600 mm and the mean annual temperature is ∼30°C. In the Anuradhapura area, paddy farming is carried out by traditional, full-time farmers and provides the main mode of income. Paddy fields are often much larger than leptospirosis-endemic areas. There are no wetlands or marshy lands in these areas, except the paddy fields during working seasons. Paddy field work depends on irrigation systems, so that between farming seasons, paddy fields become completely dry (Figure 1). The soil structure, water quality and ecological systems in Anuradhapura contrast with those of the wet zones of Sri Lanka where leptospirosis is endemic.

**Figure 2:**
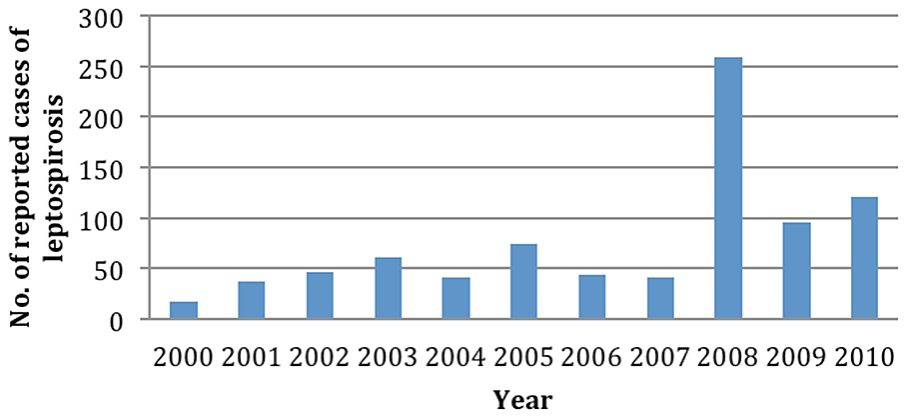
Suspected cases of leptospirosis reported to the regional epidemiologist Anuradhapura during 2000–2012 period.

In January, 2011, there was a 7-fold increase in rainfall compared to the previous year, with flooding in Anuradhapura district affecting nearly 300,000 people [2]. In 2011, after two weeks of massive flooding in Anuradhapura, physicians from Anuradhapura reported an increase in the number of febrile patents with liver and renal complications. Based on clinical presentation alone this cluster of suspected cases was considered to be an outbreak of leptospirosis [3]. There were 18 suspected leptospirosis cases reported from the Teaching Hospital Anuradhapura (THA) in January rising to 82 clinically suspected cases in February-more than the total number of suspected cases from the whole district during the eight year period, 2000–2007 (Figure 2). This represents the first large reported outbreak of leptospirosis in a non-endemic area in Sri Lanka.

Because the clinical presentation of these suspected cases was different from that reported previously, some clinicians challenged the diagnosis of leptospirosis-especially since only a few cases could be diagnosed serologically, i.e. by microscopic agglutination test (MAT). However the performance of the gold standard serological test for leptospirosis-the MAT-was limited because only genus-specific antigen (*Leptospira biflexa* serovar Patoc) was used for detection of anti-leptospiral antibodies. In the present study we use a recently published qPCR assay [4] to attempt to confirm that this outbreak of febrile illness with renal and liver complications was indeed due to leptospirosis, and if so, to quantify the bacterial load and determine whether this flood-associated outbreak was caused by the same strains implicated in the island-wide outbreak of 2008 [5].

## Methods

### Enrollment

We carried out a prospective hospital based study in THA from the 21^st^ of February to the 12^th^ of May 2011. The heavy rains continued until the first week of February resulting in most of the paddy fields in the area become partially submerged even during the harvesting period which extended to April. The appearance of new leptospirosis cases declined after the first week of May (around 3–4 weeks after the end of the harvesting period).

THA is the only tertiary care institute in the district of Anuradhapura, Sri Lanka. It has four general medicine wards with an average of 1500 admissions per month. The study population included all febrile patients >13 years of age with probable leptospirosis admitted to medical wards of THA. Probable cases of leptospirosis were identified using clinical criteria as outlined by the epidemiology unit of Sri Lanka (Table 1) [6]. Based on previous observations [5], this definition was relaxed to include “any patient suspected as having leptospirosis” by a treating physician. The protocol and case definitions for patient recruitment, sample collection and molecular methods were designed to be consistent with the protocol used during previous outbreak investigation in 2008 [5].

**Table 1:** Inclusion criteria for probable cases of leptospirosis, during the 2011 post-flood outbreak of leptospirosis in Anuradhapura, Sri Lanka.

Inclusion criteria
1. Presenting complain - acute febrile illness (fever less than 5 days) with Headache, Myalgia and Prostration, associated with any of the following signs (at least one):
• Conjunctival suffusion/conjunctival haemorrhage
• Meningeal irritation
• Anuria or oliguria/proteinuria/haematuria
• Jaundice
• Haemorrhage
• Purpuric skin rash
• Cardiac arrhythmia or failure
2. Any febrile patient who is clinically suspected as having leptospirosis, without conforming to surveillance case definition

All suspected patients admitted to THA during the study period were screened using a clinical data checklist on admission to the ward and throughout the hospital stay. Socio-demographic data and exposure history were obtained using an interviewer-administered questionnaire. A data extraction sheet was used to collect laboratory and other investigation data from patient records. In this study, we used a published qPCR assay validated previously using Sri Lankan samples [4]. A total of 5 mL of venous blood was obtained from suspected cases following standard procedures. Samples were sent to the Faculty of Medicine, Rajarata University of Sri Lanka within two hours of collection. Serum was separated; serum and whole blood samples were stored at −20°C until analyzed [7].

### Preparation of standard curve

For use in preparing standard curves (with spiked bacteria) and negative controls, venous blood was collected from a healthy individual. Exponential-phase *L. interrogans* serovar Copenhageni strain L1-130 [8] cultured in liquid EMJH media was inactivated in 10% formalin for 15 minutes then counted in a Petroff-Hausser counting chamber (Hausser Scientific). Known numbers of live *L. interrogans* L1-130 were then spiked into whole blood or serum and diluted to give final concentrations of 10^0^ to 10^8^ *Leptospira*/mL. DNA was extracted from spiked samples as described below. For *Leptospira*-negative controls, unspiked whole blood or serum from the same healthy individual was extracted as described.

#### DNA extraction

DNA was extracted from 100 µL of spiked (or unspiked) whole blood or serum using the DNeasy Blood and Tissue Kit (Qiagen) according to manufacturer's directions. Following extraction, DNA was eluted in 200 µL buffer AE in two elution steps (100 µL each) and stored at −20°C prior to use.

#### qPCR primers and probes

qPCR was performed according to a previously published protocol using the primer pair “16S Taqman_f (GAGTTTGGGAGAGGCAAGTGGAATTCCA)” and “16S Taqman_r (CGCTTTCGTGCCTCAGCGTCAGTTTTA)” and probe “16S Taqman probe1” labelled with the fluorescent reporter dye FAM (6-carboxyfluorescein) at the 5′ end, and Black Hole quencher one (BHQ-1) at the 3′ end. These pathogen-specific primers amplify a conserved region within the 16S gene of infectious leptospiral species.

### qPCR conditions and identification of positive samples

PCR reaction mixes were prepared using the iQ Supermix (Biorad) with final primer and probe concentrations of 0.5 µM and 0.2 µM, respectively, and 5 µL DNA (samples/standard curves and *Leptospira*-negative controls) or no-template control in a total reaction volume of 20 µL. Samples were amplified using a previously published qPCR assay [8]. All reactions were performed in triplicate. Fluorescence was measured at the end of the each cycle. The cycle threshold was set to two standard deviations above the mean fluorescence value for the first three cycles. A positive PCR was defined when all three replicates had a fluorescence signal above threshold. Reactions with one or two positive replicates were repeated and confirmed (or labeled as not-detected). All positives had a quantitative signal within the linear part of the standard curve greater than the cycle threshold.

### Single-tube nested PCR (STNPCR) and sequencing

All qPCR positive samples were amplified using a previously published nested PCR protocol [5]. This single-tube nested PCR was used to amplify a region of the 16S ribosomal DNA gene specific for pathogenic and intermediate *Leptospira* spp. The PCR primers were rrs- outer-F (5-CTCAGAACTAACGCTGGCGGCGCG-3′), rrs- outer-R(5′-GGTTCGTTACTGAGGGTTAAAACCCCC-3′), rrs-inner-F (5′-CTGGCGGCGCGTCTTA-3′), and rrs-inner-R (5′-GTTTTCACACCTGACTTACA-3′). PCR products were purified using the USB ExoSAP-IT PCR product cleanup kit (Affymetrics) according to manufacturer's instructions. At least two forward and two reverse sequencing reads were obtained for all samples; sequences were assembled and aligned using Geneious software then the alignments trimmed to yield a 443 bp region for phylogenetic analysis [positions 89–531 of the *rrs* gene of *L. interrogans* serovar Lai strain 56601 (NC_004342) for phylogenetic analysis. For species identification, maximum likelihood trees with support from bootstrap 500 replicates were created in MEGA5.

### Statistical analysis

Data were analyzed using SPSS version 17. All categorical data were presented as proportions. Statistical tests for categorical data were done using chi-square test.

### Ethics statement

This study conformed to the Helsinki Declaration and to local legislation. All participants gave written informed consent to participate in this study. Ethical clearance was obtained from the Research and Ethics Committee, Faulty of Medicine and Allied Sciences, Rajarata University of Sri Lanka.

## Results

### Demographic information of the study sample

**Figure 3:**
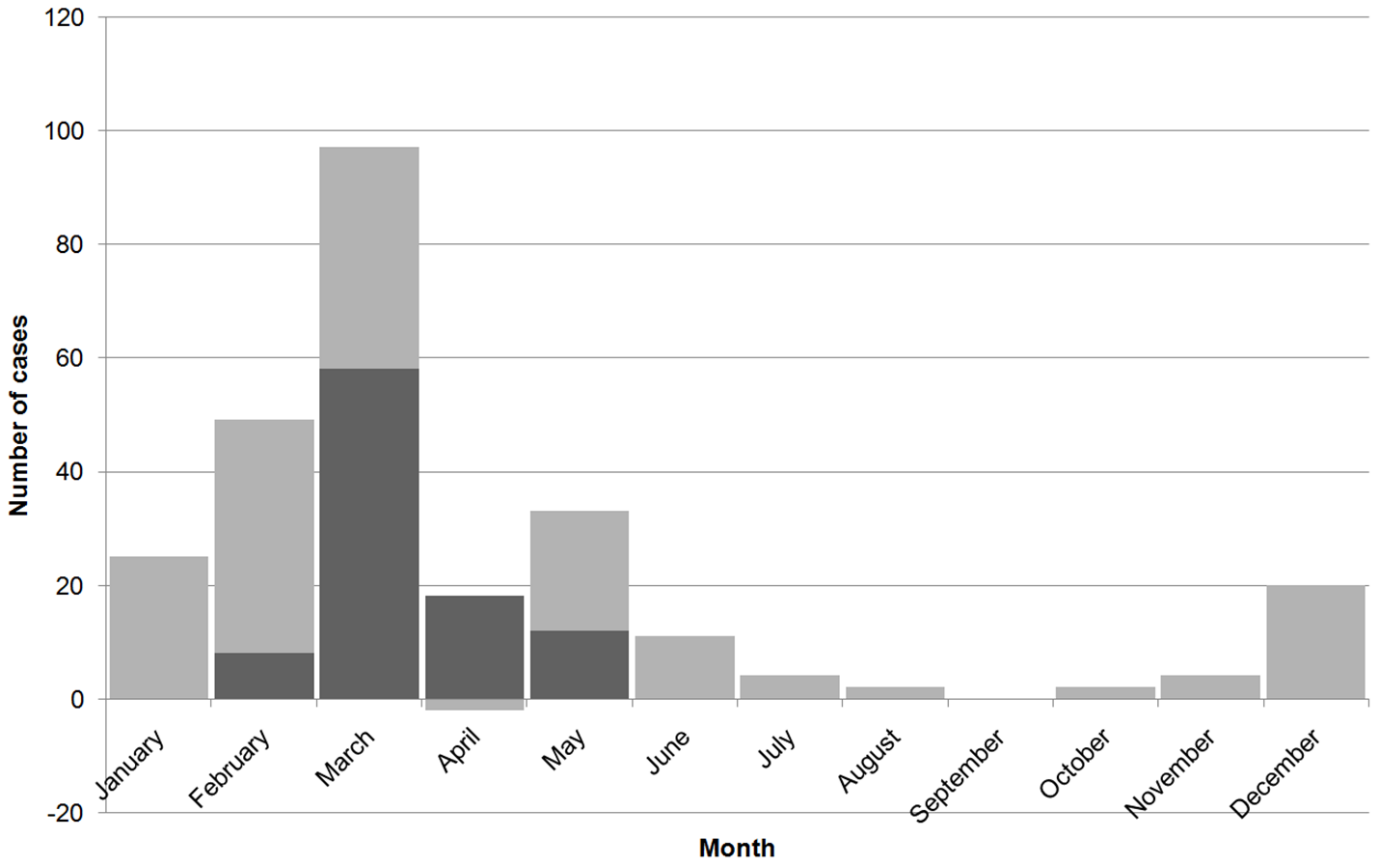
Epidemic curve of post flood leptospirosis outbreak in Anuradhapura, based on the notified cases and the number of cases recruited for the present study (in dark color).

From 21^st^ of February to 12^th^ of May 2011, 96 probable leptospirosis cases were enrolled (Figure 3). Of these probable cases, 61(63.5%) were adult males. The mean age of the study sample was 40 years (Standard deviation (SD) +/−12 years). None of these patients were admitted directly to the medical wards of THA. Before coming to THA, all 96 patients sought health care from either private (33, 34.3%) or from other government facilities. These 96 cases were from 14 administrative divisions (of the total of 20) showing a large scale rather than a focal outbreak. The median duration of fever on admission was four days (interquartile range; IQR 3–7). Paddy farmers accounted for 63.5% of the study sample.

### Disease confirmation, bacterial load and strain identification

**Figure 4:**
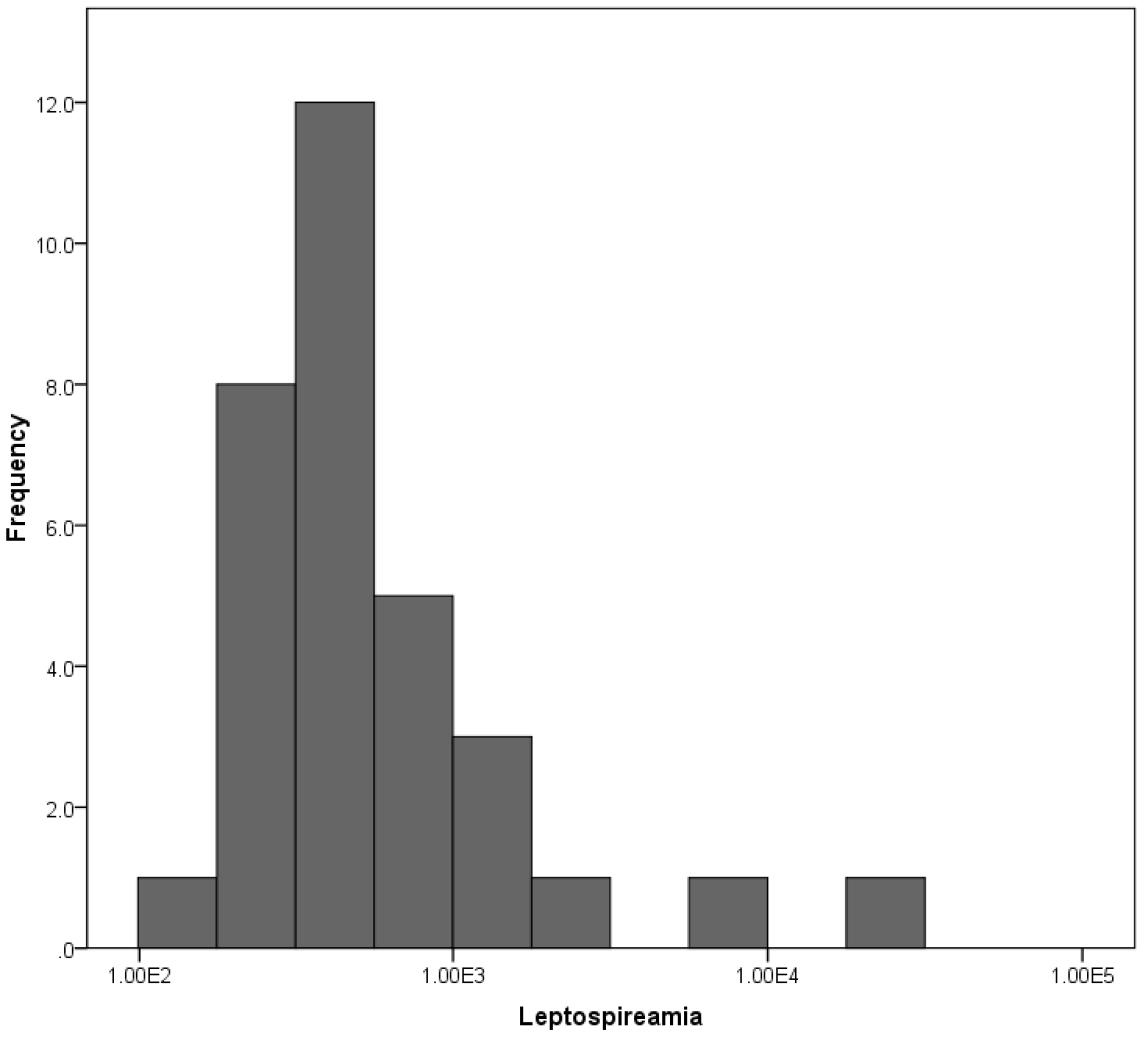
Distribution of Leptospira burden among 32 qPCR positive patients.

**Figure 5:**
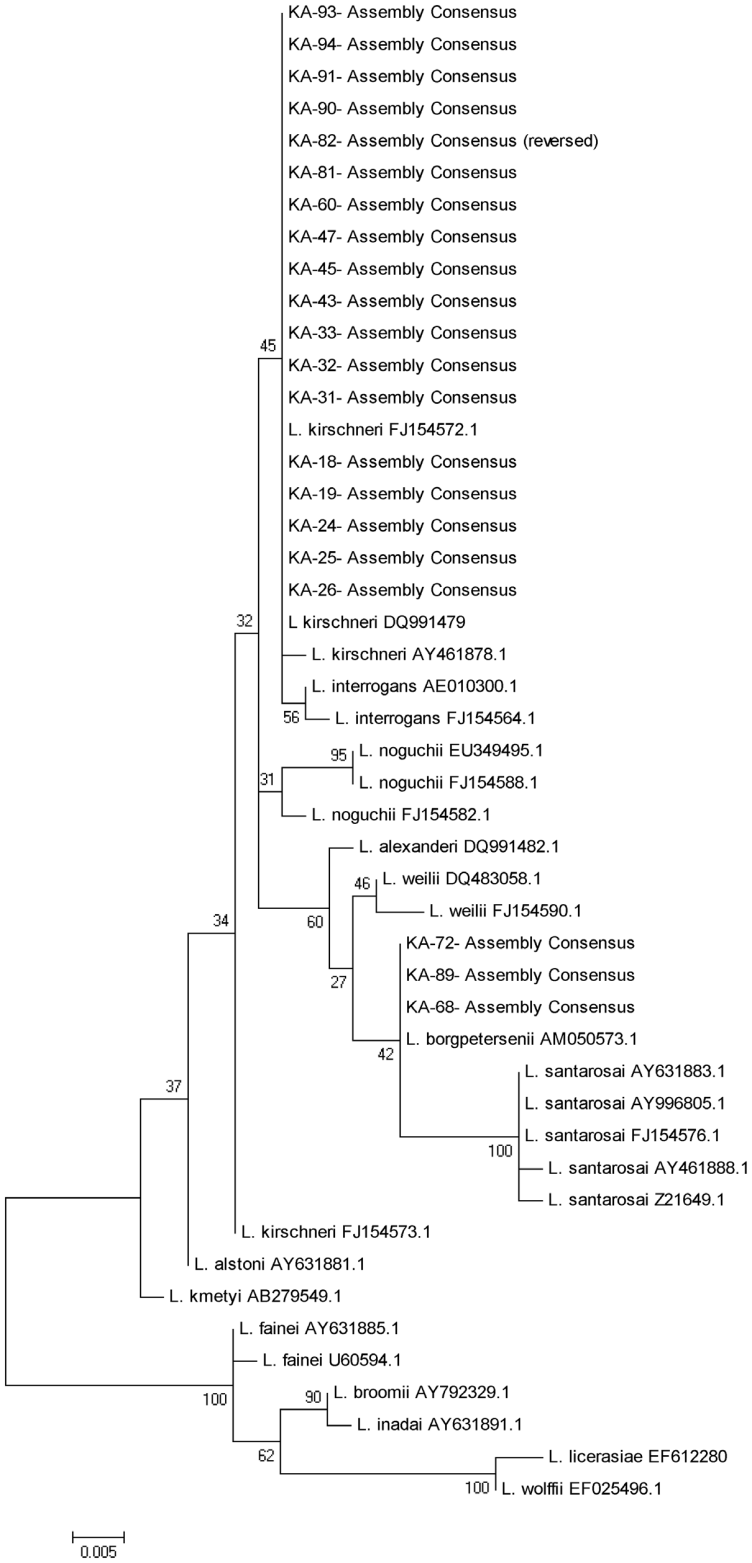
Phylogenetic tree based on the region of leptospira rrs gene. The sequences were aligned in MEGA4 using CLUSTALW, and phylogenetic distances were calculated in MEGA4 using the maximum likelihood. Numbers of nodes were bootstrap support after 500 replicates.

Of the 96 clinically suspected cases of leptospirosis, 32 (33%) were confirmed using qPCR. Bacterial load in serum/blood ranged from 10^2^ to 10^4^ *Leptospira*/mL among 32 positive cases (Figure 4). Median bacterial load was 4.1×10^2^ *Leptospira*/mL (inter-quartile range 3.1–6.1×10^2^/mL). The 16S rRNA gene could be amplified and sequenced from 26 (81.3%) of these qPCR-positive samples. Based on phylogenetic analysis of the 16S rRNA gene, *L. kirschneri* was the most common cause of disease among outbreak cases (n = 20), with strains belonging to *L. borgpetersenii* and *L. interrogans* also identified (Figure 5).

### Exposure

All 32 confirmed cases reported exposure to either a single (14, 43.8%) or multiple sources (18, 46.2%) of natural/man-made water catchments during the three-week period prior to the onset of disease (Table 2). Direct exposure to floodwater was reported by seven cases. 27 (84.4%) were engaged in paddy farming activities and all 27 reported farming as the main income source for their families. Regular exposure in paddy fields (>2 days per week) during this period was reported by 22 (67.8%) confirmed cases. Though frequent exposure in paddy farms was slightly more common in confirmed cases, the difference was not statistically significant. The presence of skin wounds was observed among 11 (34.4%) confirmed cases. However, 23 (71.9%) of the confirmed cases had skin breaches in the form of cracks around the heel, which was equally common among suspected (unconfirmed) patients.

**Table 2:** Exposure to probable sources of Leptospira during the period of three weeks prior to the onset of fever, 33 confirmed cases of leptospirosis during 2011 post-flood outbreak in Anuradhapura, Sri Lanka.

Exposure	n	%
Paddy field	27	84.4
Drains	14	43.8
Manmade running water sources	11	34.4
Natural running water	5	15.6
Other water sources	10	31.3
Flooded area	7	21.9
Marshy land	6	18.8
Animal handling	5	15.6

### Clinical profile

The median duration of fever among confirmed cases was 6 days (IQR 2–8 days) compared to 7 days (IQR 2–10 days) among probable cases. This difference was not statistically significant. Median duration of hospital stay among confirmed cases was 5 days (IQR 3–6 days). All patients had fever and headache. Of the symptoms/signs included in the proposed WHO surveillance case definition, oliguria was the most common symptom (Table 3).

**Table 3:** Clinical features of 32 qPCR confirmed cases of leptospirosis during 2011 post-flood outbreak in Anuradhapura, Sri Lanka.

Symptom/sign	n	%
Fever	32	100
Headache	32	100
Myalgia	26	81.2
Prostration	13	40.6
Oliguria	11	34.4
Diarrhea	7	21.9
Dyspnea	7	21.9
Hypotension	6	18.8
Photophobia	5	15.6
Jaundice	4	12.5
Bleeding manifestations	4	12.5
Pharyngitis	4	12.5
Bradycardia	2	9.4
Anuria	1	6.3
Hepatomegaly	1	3.1
Convulsions	1	3.1

### Sequelae

Of the 32 confirmed cases, seven (21.9%) had acute renal failure as confirmed by serum creatinine >1.5 mmol/L; two had ultrasound evidence of acute renal parenchymal disease. In addition, 17 (53.1%) cases had elevated blood urea (>40 mg/dL). Elevated liver enzymes (SGPT/SGOT) were observed in 15 (46.9%) and serum bilirubin >2 was recorded for 6 cases. Of five (15.6%) patients with myocarditis, four had ECG changes and two were confirmed by echocardiogram; three of them required inotropic drugs and two required positive pressure ventilation. There were no fatalities.

### Laboratory findings of confirmed cases

Leukocytosis of more than 11,000/mL was observed in nine (28.1%) cases, and in six (18.8%), leukopenia of less than 4000/mL was observed. All confirmed cases had a normal neutrophil count. Thrombocytopenia (<150,000/µL) was observed in 25 (78.1%) patients during the course of illness; in five (15.6%), platelet count was less than 20,000/µL.

## Discussion

In this study we demonstrate important principles of a combined public health and clinical approach to the identification and management of a difficult-to-diagnose neglected tropical disease in a resource-poor setting. A large outbreak of leptospirosis occurred in 2010 in the district of Anuradhapura, Sri Lanka, a notably dry zone of Sri Lanka where an unusually rain-associated flooding preceded the outbreak. The context of this outbreak, different from the vast experience of leptospirosis in Sri Lanka that occurs in wet zones associated with paddy farming, impeded clinical diagnosis and public health intervention. In the two and half months period extending from third week of February 2011, because of our previous experience and molecular diagnostic infrastructure development, we confirmed 32 of 96 (33.3%) clinically suspected cases using a previously published qPCR assay, which demonstrated that bacteria load in patients ranged from 10^2^ to 10^4^ *Leptospira*/mL. This outbreak, the first reported in this so-called dry zone in Sri Lanka, was characterized by atypical clinical and biological features of leptospirosis.

In contrast to previous outbreak reports, we found that most cases in the 2011 outbreak in Anuradhapura were caused by strains belonging to *L. kirschneri*, not previously known to be a common cause of human leptospirosis in Sri Lanka. Acute renal failure and myocarditis were common in the study population and the proportion of patients with complications was higher than the previous outbreak (Table 4). Renal failure and myocarditis were confirmed among 21.9% and 15.6% cases compared to 14.8% and 7.1% in 2008 outbreak. In addition, 53.1% of the patient had high blood urea nitrogen showing higher rate of acute kidney injury.

**Table 4:** Comparison of selected features of 2008 outbreak and 2011 outbreak of leptospirosis in Sri Lanka.

Feature	2008 outbreak	2011 outbreak
Outbreak	Island wide (mainly in wet zones)	Anuradhapura district (dry zone)
Period	Throughout the year	Following heavy rains and floods in first quarter of the year
Predominant species	*L. interrogans* (20/26)	*L. kirschneri* (26/32)
Median duration of fever (IQR)	6 (4–8)	6 (2–8)
Median bacterial load (IQR) *Leptospira*/mL	9.5×10^3^ (4.6×10^3^–4.9×10^4^)	4.1×10^2^ (3.1–6.1×10^2^/mL)
Renal failure (%)	13.8	21.9
Myocarditis (%)	10.3	15.6
High serum urea (%)	49.3	53.1
Thrombocytophenia (%)	47.0	78.1
Elevated liver enzymes (%)	29.2	46.9
Elevated serum bilirubin (%)	4.2	18.8

In our previous studies using nearly identical study protocols including the laboratory tests to confirm leptospirosis [5], the observations regarding infecting *Leptospira* species and serovar were different from the present study. Previous outbreak reports from Kandy, Matale and Kegalle show *L. interrogans* to be responsible for the vast majority of human leptospirosis in Sri Lanka [4], [5], [9]. Although, *L. interrogans* has historically been the pre-eminent cause of human leptospirosis in Sri Lanka [10], [11], [12], isolates of *L. borgpetersenii* serovar Ceylonica were reported in 1964 [13] and *L. kirschneri* serovar Ratnapura was isolated in 1966 from a patient and subsequently from buffalo and cattle [11]. *L. kirschneri* has not been a reported cause of human leptospirosis in Sri Lanka since these original reports in the 1960s. However, because of its location in the dry zone, no recent studies have included the district of Anuradhapura. No studies are also available from this region to show the presence of leptospirosis disease or *Leptospira* species/serovars affecting livestock or other animals in the region.

Severe complications were more common in the current study than reported previously. During the 2008 outbreak investigation thrombocytopenia and leukopenia were uncommon. Acute renal failure was observed among 14.8% of the confirmed cases and myocarditis among 8.1%. In the present study, ARF was high and also more than 50% of the confirmed cases had renal function impairment. Liver failure was not observed, but more than 50% of confirmed cases had elevated liver enzymes. In the previous report range of bacterial load was up to 10^6^/mL that was much higher than the present study. Whether these differences are due to strain or host factors is unknown. But, is noteworthy that while *L. interrogans* was the confirmed cause of 27/29 infections in the 2008 outbreak, the majority of cases reported here were caused by *L. kirschneri*. However, in the 2008 outbreak report, inclusion criteria were slightly less stringent resulting in the inclusion of a higher proportion of mild to moderate cases [5]. Thus, the rates of severe complications reported here might be partially due to the particular selection bias.

One another concern about these marked variation in *Leptospira* strain and the clinical disease is whether this is due to microgeographical variations. It has been shown for other disease like malaria [14], [15] and schistosomiasis [16], [17], [18] that microgeography may have a major influence of disease epidemiology. Geochemistry is well described as a major contributory factor in human health [19]. The previous reports are from the central, hilly, wet zones of the country, which is only around 100 km away from the present study site. However, the microgeography of these two areas are different in relation to elevation, rainfall, temperature, soil structure, crop and the ecology. Despite the extensive literature available on leptospirosis, studies on the microgeographic variation of *Leptospira* is scarce. We recommend further studies on this aspect based on our preliminary findings, which would be useful in disease control and prevention strategies.

This study shows that the unusual clinical features observed during the 2011 leptospirosis outbreak in Sri Lanka could be due to uncommon *L. kirschneri* strains that arose in the context of dry rather than wet season epidemiology and perhaps due to changing human-animal interactions or introduction of novel *Leptospira* to the region. Whether these differences are due microgeographical variations of *Leptospira* strains or due to changing populations of reservoir animals necessitates further investigation.

## Supporting Information

Checklist S1 **STROBE checklist.**
(DOC)
